# Elevated Midkine Serum Levels Are Associated with Long-Term Survival in Critically Ill Patients

**DOI:** 10.3390/ijms25010454

**Published:** 2023-12-29

**Authors:** Philipp Hohlstein, Samira Abu Jhaisha, Eray Yagmur, Dennis Wawer, Maike R. Pollmanns, Jule K. Adams, Theresa H. Wirtz, Jonathan F. Brozat, Lukas Bündgens, Karim Hamesch, Ralf Weiskirchen, Frank Tacke, Christian Trautwein, Alexander Koch

**Affiliations:** 1 Department for Gastroenterology, Metabolic Disorders and Intensive Care Medicine, RWTH-University Hospital Aachen, Pauwelsstraße 30, 52074 Aachen, Germany; phohlstein@ukaachen.de (P.H.); sabujhaisha@ukaachen.de (S.A.J.); dennis.wawer@rwth-aachen.de (D.W.); mpollmanns@ukaachen.de (M.R.P.); jadams@ukaachen.de (J.K.A.); thwirtz@ukaachen.de (T.H.W.); jonathan-frederik.brozat@charite.de (J.F.B.); lbuendgens@praxis-buendgens.de (L.B.); khamesch@ukaachen.de (K.H.); ctrautwein@ukaachen.de (C.T.); 2 Institute of Laboratory Medicine, Western Palatinate Hospital, 67655 Kaiserslautern, Germany; eyagmur@westpfalz-klinikum.de; 3 Department of Hepatology and Gastroenterology, Charité-Universitätsmedizin Berlin, Campus Virchow-Klinikum (CVK) and Campus Charité Mitte (CCM), Augustenburger Platz 1, 13353 Berlin, Germany; frank.tacke@charite.de; 4 Institute of Molecular Pathobiochemistry, Experimental Gene Therapy and Clinical Chemistry (IFMPEGKC), RWTH-University Hospital Aachen, Pauwelsstraße 30, 52074 Aachen, Germany; rweiskirchen@ukaachen.de

**Keywords:** Midkine, intensive care unit, critical illness, sepsis, biomarker, human, inflammation, immune system, prognosis, survival, mortality

## Abstract

Midkine (Mdk) is a multifunctional protein involved in inflammatory processes. Hence, circulating Mdk is increased in sepsis and has been previously suggested as a potential biomarker in these patients. The aim of this study was to elucidate the role of Mdk serum concentrations in critical illness and sepsis and to verify its value as a prognostic biomarker. Thus, we analyzed the Mdk serum concentrations of 192 critically ill patients on admission to the medical intensive care unit (ICU). While the serum levels of Mdk at admission were similar in septic and nonseptic critical illness (362 vs. 337 ng/L, *p* = 0.727), we found several interesting correlations of Mdk to laboratory and clinical markers associated with ischemia or hypoxia, e.g., to renal failure and hepatic injury. Mdk serum concentrations at admission did not differ between various causes of sepsis or other critical illness. Most noticeable, we observed upregulated Mdk serum concentrations at admission in patients surviving in the long-term, which was only seen in nonseptic critical illness but not in sepsis. Our study suggests a relevant role of Mdk in critically ill patients in general and highlights the possible protective features of Mdk in critical illness.

## 1. Introduction

In internal and critical care medicine, predicting the patient outcome and diagnosis of sepsis and septic shock remain major challenges with ongoing need for research. Mortality is generally high in patients in the intensive care unit, with around 30 to 50% mortality in sepsis [1]. There is still a scarcity of routinely and reliably applicable laboratory markers indicating sepsis and critical illness or their prognosis. Specifically, sepsis is constituted by a life-threatening host immune response leading to severe organ dysfunction [2]. This organ dysfunction is defined and measured using the SOFA (sepsis-related organ failure assessment) score [3]. However, the exact mechanisms behind the uncontrolled infection, the dysregulated immune response and the development of organ dysfunction are still not clearly understood, nor can be exactly measured, and thus warrant further investigation [4,5].

Midkine (Mdk), known as the neurite growth-promoting factor 2, is a multifunctional protein that was first discovered in mouse embryogenesis [6,7]. Along with pleiotrophin (PTN), it is part of a structurally unique family of heparin-binding growth factors [8]. Mdk is a 13 kDa cysteine-rich polypeptide consisting of two domains (N- and C-domains) held together with disulfide bridges [8,9]. Moreover, Mdk is a soluble and secreted protein and could therefore serve as a biomarker for disease [10]. While Mdk gene expression has been detected at many sites, including the gastrointestinal tract, spleen, kidney, lungs and thyroid gland, its strongest relative expression seems to be in the mucosa of the small intestine [10,11,12,13,14,15,16,17]. Despite this Mdk gene expression, there is mostly a lack of any corresponding detectable Mdk protein expression in healthy tissues as opposed to malignant tissue [18,19]. Notably, the only healthy tissue with consistent Mdk protein expression seems to be the kidney [20].

Mdk has been shown to be overexpressed in various disease processes involving inflammation, most prominently in malignant diseases, including at least 20 different cancer types [10,21]. However, the alteration of Mdk has also been described in ischemic disease [22,23,24,25], kidney injury [24,26,27,28,29,30] and autoimmune disease [31,32,33,34,35,36]. Apart from its function as a growth factor, Mdk exerts numerous biological functions in the inflammation process and the recruitment of inflammatory cells [8,12,31,33,37,38,39], as well as the preservation of tissue viability during hypoxic stress [40]. Additionally, a strong antibacterial activity of Mdk has been demonstrated in vitro [8,41]. Hence, its involvement in the emergence and pathophysiology of sepsis can be anticipated. In fact, in a pilot study from 2010 consisting of 38 septic patients, 82 patients with active inflammatory bowel disease (IBD) and 87 healthy subjects, Mdk was increased in septic and IBD patients [42]. Another small study from 2020 involving 26 septic patients demonstrated increased plasma Mdk levels in sepsis survivors compared to non-survivors at day 28 [43]. Apart from the elevation in human sepsis, the inhibition of Mdk has also been demonstrated to ameliorate sepsis-induced lung injury in a mouse model in a recent study from 2021 [44]. Besides these studies, little is known about the involvement of Mdk not only in sepsis but in critical illness in general. Therefore, we conducted a detailed clinical study investigating of the regulation of Mdk in critical illness and sepsis, its association with various clinical markers and organ dysfunction and its potential as a prognostic biomarker.

## 2. Results

### 2.1. Midkine Serum Concentrations Do Not Differ between Critically Ill Patients with and without Sepsis

**Figure 1:**
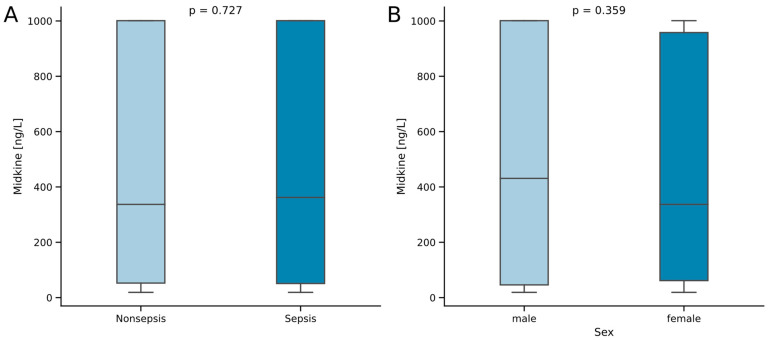
Serum Midkine concentrations in critically ill patients with and without sepsis (**A**) and comparison between sexes (**B**). Sample sizes: patients *n* = 192, nonsepsis *n* = 67, sepsis *n* = 125. Significance between groups was assessed using the Mann–Whitney U test. *p*-values < 0.05 were considered statistically significant.

The cohort of this study comprises 125 patients admitted to the medical ICU due to sepsis and 67 patients admitted due to other critical illness. The median age of the cohort was 64.5 years, without statistical difference between septic and nonseptic critically ill patients. No differences were observed between the two study groups for age, sex, comorbidities (measured using the Charlson Comorbidity Index) or mortality. Notably, we also did not observe a significant difference in the levels of Mdk between the two groups (Figure 1A). However, patients with septic disease showed higher scores for disease severity (APACHE II, median of 18 vs. 16 points, *p* = 0.039) and organ insufficiency (SOFA, median of 11 vs. 7 points, *p* = 0.006). Coherently, patients with sepsis had higher demands for mechanical ventilation (73.6 vs. 57.5%, *p* = 0.036), as well as vasopressor therapy (70 vs. 47.4%, *p* = 0.005), and thus required a longer stay in the ICU (median of 10 vs. 6 days, *p* < 0.001; Table 1). Concerning other possible influence factors of Mdk serum levels, we did not observe a difference between sexes (Figure 1B) nor a correlation with age or body mass index (BMI) (Table 2).

### 2.2. Midkine Serum Levels Are Not Associated with Disease Etiology in Critically Ill Patients

In this study, most sepsis patients were treated due to a pulmonary focus (55.2%). Other sites of infection were the abdomen (15.2%) or the urogenital tract (8%). Other sepsis patients (21.6%) were treated due to bloodstream infections, skin infections or an unknown focus of infection. Patients with nonseptic critical illness were treated due to cardiocirculatory disease (19.4%), advanced liver disease (19.4%) or respiratory failure (14.9%), as well as numerous other diseases (46.3%). Further, looking into the potential regulation between those disease etiologies, we observed a higher level of Mdk in pulmonary and other focuses (median of 425 and 431 ng/L, respectively), as compared to abdominal or urogenital infections (median of 105 and 270 ng/L, respectively), although those changes did not reach a level of statistical significance (*p* = 0.481). Nonseptic patients did not show a regulation of Mdk in different disease etiologies (*p* = 0.772, Table 3).

To examine the influence of preexisting comorbidities on the serum levels of Mdk, we compared Mdk serum levels in patients with and without various diseases. Here, we could not show any differences in Mdk serum levels for diabetes, liver disease, coronary artery disease, hypertension, chronic alcohol abuse, chronic obstructive lung disease or active malignancy (Table 4).

### 2.3. Midkine Correlates with Clinically Established Biomarkers of Bacterial Inflammation, Kidney Function, Coagulation Function and Insulin Metabolism

Next, we aimed to evaluate other potential factors regulating Mdk serum concentrations in critical illness. For further investigation, we performed extensive correlation analyses between Mdk serum concentrations and a wide selection of laboratory as well as clinical markers. Concerning peripheral blood counts and inflammatory markers, we observed a positive correlation of medium strength between Mdk and procalcitonin (Spearman’s r = 0.263, *p* = 0.001). However, such a correlation was not seen for other inflammatory markers such as peripheral leucocyte count or the C-reactive protein (CRP). Furthermore, Mdk also shows weak to medium positive correlations to the markers of kidney dysfunction, i.e., uric acid (Spearman’s r = 0.190, *p* = 0.018), creatinine (Spearman’s r = 0.197, *p* = 0.006) and cystatin C (Spearman’s r = 0.231, *p* = 0.010). For the markers of hepatobiliary injury, we detected a moderate correlation of Mdk to aspartate aminotransferase (AST, Spearman’s r = 0.256, *p* = 0.001) and alanine aminotransferase (ALT, Spearman’s r = 0.154, *p* = 0.034), while other markers of liver function such as bilirubin or the internationalized normalized ratio (INR) did not correlate with Mdk. Interestingly, we also observed a moderate correlation with the activated partial thromboplastin time (aPTT, Spearman’s r = 0.330, *p* < 0.001). While the markers of the cardiocirculatory system or ICU parameters did not correlate to Mdk serum levels, we noted correlations to the markers of metabolism. Here, insulin (Spearman’s r = 0.229, *p* = 0.031) and C-peptide (Spearman’s r = 0.254, *p* = 0.016) levels showed a medium positive correlation to Mdk serum levels (Table 3).

### 2.4. Midkine Predicts Long-Term Survival in Critically Ill Patients

**Figure 2:**
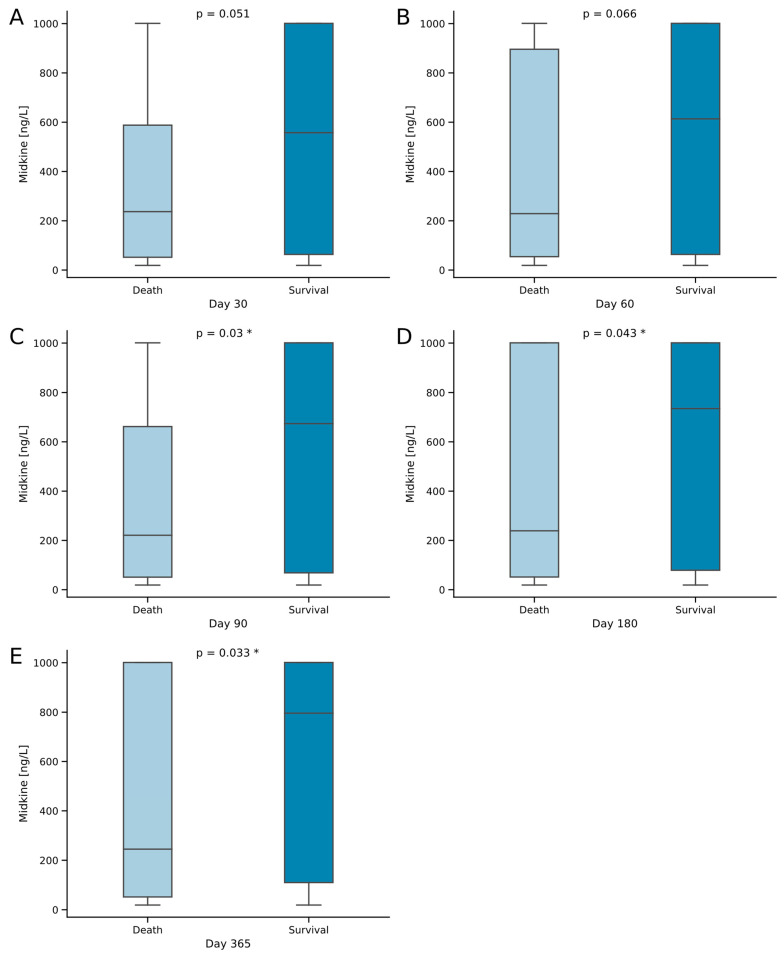
Midkine levels in a consecutive survival analysis of critically ill patients treated in the ICU. (**A**–**E**) Survival status on days 30 through 365. Sample sizes: patients *n* = 192. * Significance between groups was assessed using the Mann–Whitney U test. *p*-values < 0.05 were considered statistically significant and were highlighted (“*”).

**Figure 3:**
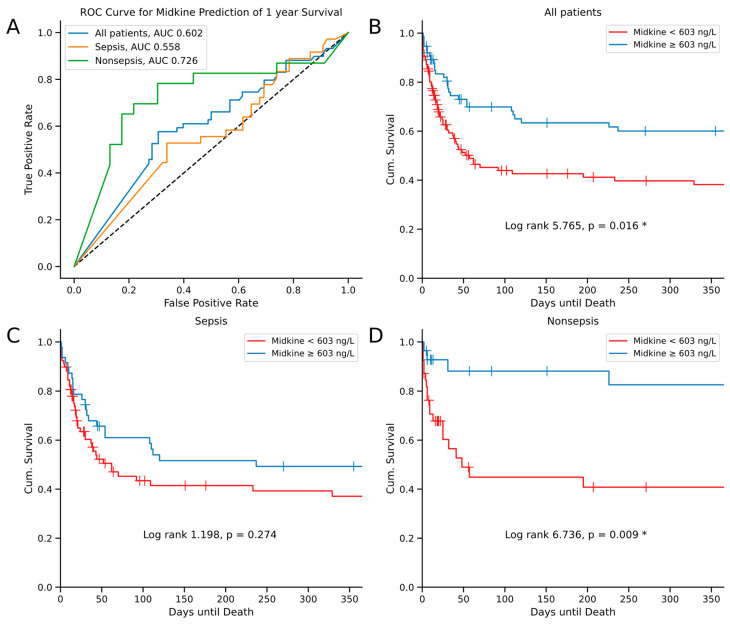
Receiver operating characteristic (ROC) curves for the prediction of one-year survival using serum Midkine levels in all patients, sepsis and nonsepsis patients. Black dashed line represents the ROC curve for a random guess (**A**). Kaplan–Meier curves for Midkine <603 ng/L (red) and ≥603 ng/L (blue) in all patients (**B**), septic patients (**C**) and nonseptic patients (**D**). Censored events are indicated with a crossing vertical line. Cut-off values of the Kaplan–Meier curve were determined using the Youden index for all patients. Sample sizes: patients *n* = 192, nonsepsis *n* = 67, sepsis *n* = 125. * Significance between groups was assessed using the log-rank test. *p*-values < 0.05 were considered statistically significant and were highlighted (“*”). Abbreviations: AUC: area under curve.

Next, we focused on elucidating the prognostic value of Mdk serum levels in critically ill patients. First, we looked at the Mdk levels at admission in comparison between surviving and deceased patients at consecutive standardized time points over one year (i.e., 30, 60, 90, 180 and 365 days). Strikingly, we observed increased Mdk serum concentrations (obtained at ICU admission) in surviving patients for all mortality time points, which reached statistical significance including day 90 and later (*p* = 0.03 at day 90, *p* = 0.043 at day 180, *p* = 0.033 at day 365; Figure 2). In a subsequent receiver operating curve (ROC) analysis, Mdk serum levels showed an area under the curve (AUC) of 0.602 for the prediction of survival at one year (Figure 3A). To understand the difference between septic and nonseptic patients, we performed the ROC analysis also for those subgroups. Here, septic patients showed a lower AUROC of 0.558 in comparison to nonseptic patients with an AUROC of 0.726 (Figure 3A). In addition, we conducted a Kaplan–Meier curve analysis with the Youden index as a means to calculate an ideal cut-off value with respect to survival prediction for Mdk serum levels at 603 ng/L at admission to the ICU. First analyzing all patients, the Kaplan–Meier curves showed the largest separation towards the end of the follow-up timeframe at day 365 (log-rank 5.765, *p* = 0.016; Figure 3B). To further dissect the insights of the ROC analysis, we also conducted separate Kaplan–Meier analyses for our study cohorts of septic and nonseptic patients. In nonseptic patients, we could show an even larger curve separation (log-rank 6.736, *p* = 0.009; Figure 3D). However, in septic patients, we did not see a statistically significant curve separation (log-rank 1.198, *p* = 0.274; Figure 3C).

## 3. Discussion

Previously, peripheral Mdk levels have been shown to be elevated in sepsis and might indicate prognosis in these patients [42,43]. In this study, we investigated the serum levels of Mdk in critical illness and sepsis along with the possible use of Mdk for the prognostication of survival. While being independent of age, sex or BMI, Mdk serum levels did not differ between critically ill patients with and without sepsis. Moreover, we did not observe any changes in Mdk between the different disease categories apart from trends towards higher Mdk in pulmonary and other sepsis. The data suggest a correlation of Mdk serum levels to the markers of bacterial inflammation, kidney function, coagulation function and insulin metabolism. Most interestingly, we reported higher levels of Mdk in patients surviving the ICU. Our findings indicate a prognostic character of Mdk in critical illness, but on the contrary, not in sepsis.

Mdk is a soluble and secreted multifunctional protein, which is most prominently known as a biomarker in cancer research [10]. Mdk has been reported as elevated in sepsis in a small Polish pilot study from 2010 [42]. Our data suggests that the elevation of Mdk seems to be a feature of critical illness, rather than just sepsis alone, as the levels of serum Mdk are similar in the groups of our study (Figure 1A). Encouraging its use as a potential biomarker, we can report the independence of Mdk serum levels from age, sex and BMI (Figure 1B, Table 2). Previous data did not suggest changes in Mdk serum levels regarding the site of infection leading to sepsis [42]. In our ICU cohort, we also did not find any statistically significant differences in circulating Mdk serum levels between the causes of sepsis (Table 3). Moreover, we also did not find differences between the categories of nonseptic disease (Table 3), continuously suggesting that Mdk elevation is a general feature of critical illness. Regarding comorbidities, Mdk is known to be elevated in malignant disease [10,21], ischemic disease [22,23,24,25], kidney injury [24,26,27,28,29,30] and autoimmune disease [31,32,33,34,35,36]. However, when comparing the occurrence of several comorbidities of critically ill patients in our cohort, we did not find alterations in peripheral Mdk serum levels dependent on the comorbidity (Table 4). This indicates overlaying factors influencing the circulating Mdk in acute illness. However, concerning the previously described involvement in the pathogenesis of Mdk in kidney injury, we describe multiple correlations of Mdk to the markers of kidney function, i.e., creatinine and cystatin C (Table 2). As a heparin-binding growth factor, Mdk is known to be increased with heparin administration [45]; likewise, Mdk was also correlated to the length of aPTT (which is increased in heparin administration) in our study but not INR (Table 2). Interestingly, we found positive correlations of Mdk to hepatic transaminases (AST and ALT; Table 2) with the absence of correlation to peripheral bilirubin, supporting the concept of elevation of Mdk in ischemic states and hypoxic stress [22,23,24,25], which is a common feature of severe critical illness. While one study did not find differences in Mdk levels in critically ill patients with and without cardiovascular, respiratory, hematologic or kidney dysfunction [42], another study described differences in Mdk dependent on the severity of acute respiratory syndrome (ARDS) and kidney injury [43]. Our data seems to fit somewhere in between the results of those studies, as we report an association between kidney injury and Mdk serum levels but no correlation to the Horovitz quotient (PaO_2_/FiO_2_) for the diagnosis of ARDS (Table 2).

Arguably the most relevant finding of our study is the association of elevated serum Mdk on admission to the ICU to increased survival of critical illness. Currently, there is conflicting evidence regarding the impact of Mdk serum levels on survival in critical illness. On one hand, many studies suggest protective biological effects of Mdk, e.g., the recruitment of inflammatory cells [8,12,31,33,37,38,39], the preservation of tissue viability in hypoxic stress [40] and the antibacterial activity of Mdk in vitro [8,41]. On the other hand, a small Chinese study from 2020 including 26 septic patients, described lower levels of circulating plasma Mdk in survivors at day 28 [43]. There is no evidence available supporting the impact of Mdk on survival in nonseptic critically ill patients. Although we also demonstrated an association of Mdk with mortality in this study, there are a few key differences to be discussed. Firstly, in our study, Mdk seems to have protective effects, as we consistently measured higher Mdk levels on admission in surviving patients for all survival analysis time points. Secondly, the data of this study described an association of Mdk to survival in the long-term, rather than in the short-term (i.e., day 28). Lastly, we found an association of Mdk levels on admission to survival in all critically ill patients, which remarkably was not retained in sepsis, but rather in nonseptic critical illness. This study supports the concept of protective effects of Mdk in critical illness. Moreover, the entry levels of Mdk could reflect the inflammatory state of the disease and therefore impact the survival of patients via the widespread cytoprotective effects of Mdk on inflammation, apoptosis and in hypoxic stress, independent from disease etiology in critical illness [10,46,47,48].

Acknowledging the limitations of our study is important. By conducting a single-center study, we were able to achieve high technical accuracy and reproducibility. Although we investigated Mdk serum levels in the context of a large cohort in a biomarker study, the extensive analyses of patient subgroups lacked the statistical power to reliably detect smaller alterations in biomarker concentrations. Possibly related to this, we detected several correlations of medium strength of Mdk with laboratory and clinical markers (Table 2), the clinical value of which must be carefully evaluated. Furthermore, the lack of Midkine measurements in healthy controls makes comparisons between healthy individuals and critical illness impossible. Moreover, the cut-off of Mdk measurements for the assay we used was 1000 ng/L. A considerable number of measurements were at this upper cut-off and therefore probably in part well above it. Measuring Mdk levels above this cut-off would most likely lead to a deeper understanding of the distribution and regulation of Mdk. In addition, follow-up measurements at later time points during the intensive care treatment would also enhance our understanding of the role of Mdk in critical illness.

## 4. Materials and Methods

### 4.1. Study Design

This study was conducted as a retrospective, observational study to elucidate the role of Mdk in critically ill patients in a medical intensive care unit (ICU). For inclusion in this study, written informed consent was attained from the patient, his or her spouse, or legal guardian. We included 192 patients admitted to our medical intensive care unit of the Department of Gastroenterology, Digestive Disease and Intensive Care Medicine. Patients with consent, who were above or equal to the age of 18 years, were included in this study, as described previously [49,50]. We excluded (a) patients with expected short-term (less than 48 h) intensive care treatment, (b) patients admitted from another ICU and (c) patients admitted due to acute poisoning. The diagnosis of sepsis was established using the Third Consensus Definition for Sepsis (Sepsis-3) [2]. Patient comorbidities were assessed using the Charlson Comorbidity Index [51]. For collecting follow-up data concerning survival of patients, we contacted the patient, his or her relatives, or primary care physician. This study was approved by the local ethics committee (EK150/06) of the RWTH Aachen University Hospital and was conducted in accordance with the 1964 Declaration of Helsinki.

### 4.2. Midkine (Mdk) Measurements

Collection of blood samples was conducted at the time of admission to the intensive care unit. Blood samples were centrifuged at 4 °C for 10 min and were aliquoted into samples of 1 milliliter before being frozen at −80 °C until further use. Mdk concentrations were measured using a commercially available ELISA in accordance with the instructions of the manufacturer (BioVendor—Laboratorni medicina a.s., Karasek 1767/1, 621 00 Brno, Czech Republic). The measurements were performed blinded to clinical or other laboratory data of patients.

### 4.3. Statistical Analysis

Data was analyzed and graphed using SPSS version 29 (SPSS, Chicago, IL, USA) and the packages NumPy version 1.21.5 [52], Pandas version 1.4.4 [53], Matplotlib version 3.5.2 [54], Seaborn version 0.11.2 [55], Pingouin version 0.5.3 [56], Scikit-learn version 1.0.2 [57] and Lifelines version 0.27.7 [58] in Jupyter Notebooks version 6.5.4 [59] using Python version 3.11 [60]. Data is given as median and range due to the possible skewed distribution of parameters. The two-tailed Mann–Whitney U test or chi-squared test was applied to two groups of unpaired samples, as normal distribution could not be assumed. The Kruskal–Wallis test was applied to more than two groups. A significance level of *p* = 0.05 was used for all corresponding calculations. The correlation of parameters was assessed using the Spearman’s rank correlation test. The Youden index (as the sum of sensitivity and specificity minus one) was calculated to identify optimal cut-off values for parameters to discriminate prognosis. To evaluate the quality of a predictive marker, receiver operating characteristics (ROC) curves and the corresponding area under the curve (AUC) were generated. Patient survival was depicted by Kaplan–Meier curves followed by a log-rank test for level of significance.

## 5. Conclusions

Our study shows that Mdk serum concentrations are similar between septic and nonseptic individuals in a large cohort of critically ill patients. Possibly linked to the states of ischemia or hypoxia, we reveal several interesting correlations of Mdk concentrations, e.g., to renal failure and hepatic injury. Most strikingly, this study associates lower Mdk serum concentrations with higher mortality in critical illness, with the strongest influence in nonseptic patients. Possible future research should aim at a deeper understanding and validation of the role of Mdk in nonseptic critical illness.

## Figures and Tables

**Table 1:** Baseline patient characteristics.

Parameter	All Patients	Sepsis	Nonsepsis	*p*-Value
Number *n*	192	125	67	
Sex (male/female) *n*	113/79	78/47	35/32	0.226
Age (years)	64.5 (18–89)	65 (21–89)	63 (18–87)	0.663
APACHE II score	17 (2–40)	18 (3–40)	16 (2–37)	0.039 *
SOFA score	10 (0–18)	11 (3–17)	7 (0–18)	0.006 *
Charlson Comorbidity index	4 (0–16)	4 (0–16)	4 (0–13)	0.297
Mechanical ventilation *n* (%)	130 (68.0)	92 (73.6)	38 (57.5)	0.036 *
Vasopressor demand *n* (%)	115 (62.2)	84 (70.0)	31 (47.7)	0.005 *
ICU days *n*	8 (1–137)	10 (1–137)	6 (2–44)	<0.001 *
Death in ICU *n* (%)	52 (27.1)	38 (30.4)	14 (20.9)	0.214
30-day mortality *n* (%)	57 (34.5)	41 (36.3)	16 (30.8)	0.606
1-year mortality *n* (%)	88 (59.9)	65 (64.4)	23 (50.0)	0.142
Midkine (ng/mL)	358 (19–1000)	362 (19–1000)	337 (19–1000)	0.727

The median and range (in parentheses) are given unless indicated otherwise. Abbreviations: APACHE: acute physiology and chronic health evaluation; SOFA: sequential organ failure assessment; ICU: intensive care unit. * Significance between sepsis and nonsepsis patients was assessed using the Mann–Whitney U test or chi-squared test, respectively. *p*-Values < 0.05 were considered statistically significant and were highlighted (“*”).

**Table 2:** Correlations of clinical and laboratory parameters with Midkine serum concentrations at ICU admission.

Parameters	r	*p*-Value
Demographics		
Age	0.005	0.943
Body mass index	0.044	0.550
Blood count and markers of inflammation		
Leukocytes	0.005	0.945
Hemoglobin	0.077	0.290
Platelets	−0.024	0.747
C-reactive Protein	0.084	0.249
Procalcitonin	0.263	0.001 *
Interleukin 6	0.035	0.670
Interleukin 10	0.054	0.594
Electrolytes and renal system		
Sodium	−0.066	0.362
Potassium	0.058	0.428
Urea	0.103	0.156
Uric acid	0.190	0.018 *
Creatinine	0.197	0.006 *
Cystatin C	0.231	0.010 *
Hepato-pancreatico-biliary system and coagulation		
Protein, total	−0.020	0.803
Albumin	−0.085	0.339
INR	−0.058	0.432
aPTT	0.330	<0.001 *
Bilirubin, total	0.013	0.862
γGT	0.127	0.082
AST	0.256	0.001 *
ALT	0.154	0.034 *
Lipase	0.103	0.211
Cardiopulmonary system		
NTproBNP	0.131	0.212
Norepinephrine demand at day 1 (µg/day)	0.055	0.456
Horovitz quotient (PaO_2_/FiO_2_)	−0.047	0.710
Ventilatory FiO_2_ demand	0.079	0.527
Net fluid balance day 1	−0.021	0.776
Net fluid balance day 3	−0.062	0.450
Metabolism		
Glucose	0.027	0.712
HbA1c	0.059	0.582
Insulin	0.229	0.031 *
C-Peptide	0.254	0.016 *
HOMA IR	0.161	0.133
Cholesterol	0.039	0.633
HDL-cholesterol	−0.128	0.237
LDL-cholesterol	−0.010	0.928
Triglycerides	0.084	0.303
Disease severity parameters		
Days on ICU	0.046	0.526
SOFA day 1	−0.037	0.749
SOFA day 3	−0.059	0.668
APACHE-II day 1	0.009	0.909
APACHE-II day 3	−0.104	0.420

Spearman’s rank correlation test was used to calculate significant correlations of positive and negative nature. *p*-values < 0.05 were considered statistically significant and were highlighted (“*”). Abbreviations: ICU: intensive care unit; INR: international normalized ratio; γGT: Gamma-glutamyl transpeptidase; ALT/AST: alanine/aspartate aminotransferase; NTproBNP: N-terminal pro B-type natriuretic peptide; FiO_2_: fraction of inspired oxygen; HbA1c: glycosylated hemoglobin A1; HOMA: homeostatic model assessment; HDL: high-density lipoprotein; LDL: low-density lipoprotein; SOFA: sequential organ failure assessment; APACHE-II: acute physiology and chronic health evaluation II.

**Table 3:** Disease etiology of the study population and subgroup Midkine concentrations.

Etiology of (Non)Septic Critical Illness	Sepsis *n* = 125, *n* (%)	Nonsepsis *n* = 67, *n* (%)	Midkine (ng/L)	*p*
Pulmonary	69 (55.2)		425 (19–1000)	0.481
Abdominal	19 (15.2)		105 (19–1000)	
Urogenital	10 (8)		270 (22–1000)	
Other	27 (21.6)		431 (19–1000)	
Cardiocirculatory disorder		13 (19.4)	365 (19–1000)	0.772
Respiratory failure		10 (14.9)	300 (19–1000)	
Advanced liver disease		13 (19.4)	337 (19–1000)	
Other		31 (46.3)	240 (19–1000)	

The absolute numbers and percentages of the respective subgroup (in parentheses) or median and range (in parentheses) are given. Significance between more than two groups was assessed using the Kruskal–Wallis test. *p*-values < 0.05 were considered statistically significant.

**Table 4:** Comorbidities and their influence on Midkine levels at ICU admission.

Comorbidity	Midkine Concentration in ng/L, Median (Range)	*p*
Diabetes (*n* = 50)	209 (19–1000)	0.182
Liver disease (*n* = 20)	225 (19–1000)	0.735
Coronary artery disease (*n* = 63)	379 (19–1000)	0.617
Hypertension (*n* = 75)	462 (19–1000)	0.162
Chronic alcohol abuse (*n* = 25)	337 (19–1000)	0.543
Chronic obstructive lung disease (*n* = 25)	425 (19–1000)	0.939
Active malignancy (*n* = 23)	151 (19–1000)	0.302

The median and range (in parentheses) are given unless indicated otherwise. Significance between groups was assessed using the Mann–Whitney U test. *p*-values < 0.05 were considered statistically.

## Data Availability

The original data sets presented in this study are available on request from the corresponding author.
